# Biological profile of monocyte-derived macrophages in coronary heart disease patients: implications for plaque morphology

**DOI:** 10.1038/s41598-019-44847-3

**Published:** 2019-06-18

**Authors:** Sonia Eligini, Nicola Cosentino, Susanna Fiorelli, Franco Fabbiocchi, Giampaolo Niccoli, Hesham Refaat, Marina Camera, Giuseppe Calligaris, Stefano De Martini, Alice Bonomi, Fabrizio Veglia, Francesco Fracassi, Filippo Crea, Giancarlo Marenzi, Elena Tremoli

**Affiliations:** 1 Centro Cardiologico Monzino I.R.C.C.S., Milan, Italy; 2 Department of Cardiovascular & Thoracic Sciences, Fondazione Policlinico Universitario A. Gemelli, I.R.C.C.S., Rome, Italy; 3 Università Cattolica del Sacro Cuore, Istituto di Cardiologia, Rome, Italy; 4 Dipartimento di Scienze Farmacologiche e Biomolecolari, Università degli Studi di Milano, Milan, Italy; 5 Institute of Cardiology, Zagazig University, Zagazig, Egypt

**Keywords:** Cell biology, Diseases

## Abstract

The prevalence of a macrophage phenotype in atherosclerotic plaque may drive its progression and/or instability. Macrophages from coronary plaques are not available, and monocyte-derived macrophages (MDMs) are usually considered as a surrogate. We compared the MDM profile obtained from coronary artery disease (CAD) patients and healthy subjects, and we evaluated the association between CAD MDM profile and *in vivo* coronary plaque characteristics assessed by optical coherence tomography (OCT). At morphological analysis, MDMs of CAD patients had a higher prevalence of round than spindle cells, whereas in healthy subjects the prevalence of the two morphotypes was similar. Compared to healthy subjects, MDMs of CAD patients had reduced efferocytosis, lower transglutaminase-2, CD206 and CD163 receptor levels, and higher tissue factor (TF) levels. At OCT, patients with a higher prevalence of round MDMs showed more frequently a lipid-rich plaque, a thin-cap fibroatheroma, a greater intra-plaque macrophage accumulation, and a ruptured plaque. The MDM efferocytosis correlated with minimal lumen area, and TF levels in MDMs correlated with the presence of ruptured plaque. MDMs obtained from CAD patients are characterized by a morpho-phenotypic heterogeneity with a prevalence of round cells, showing pro-inflammatory and pro-thrombotic properties. The MDM profile allows identifying CAD patients at high risk.

## Introduction

Macrophages are heterogeneous in morphology and in function, and this heterogeneity has been well documented in different physiological and pathological conditions^1–3^. Also in experimental models and human atherosclerotic lesions, macrophages display morpho-phenotypic heterogeneity, with distinct subpopulations showing pro-inflammatory or reparative properties^1,4–7^. On these premises, it has been hypothesized that the prevalence of a specific macrophage phenotype may exert harmful or beneficial functions in the progression and/or destabilization of the atherosclerotic plaque^8,9^. We have previously reported that monocytes isolated from healthy subjects and spontaneously differentiated into macrophages (MDMs) give rise to two dominant morphotypes coexisting in the same culture, namely round MDMs showing a non-inflammatory and reparative phenotype, and spindle MDMs exhibiting a pro-inflammatory profile^10^. To date, no information is available concerning the behavior of MDMs in patients with coronary artery disease (CAD). Therefore, an insight into the signature of MDMs obtained from patients with established CAD and their potential association with *in vivo* coronary plaque features might be of help in the understanding of the pro/anti-atherogenic potential of these cells.

Optical coherence tomography (OCT) provides high-resolution (10 µm) *in vivo* images of the atherosclerotic plaque, allowing to acquire detailed information about its morphology and composition, including fibrous cap thickness, lipid core, and macrophage accumulation. Combining plaque characterization at OCT investigation with morpho-phenotypic data of MDMs may potentially provide a unique signature, in terms of MDM morpho-phenotype, for the identification of those plaques that are most likely to lead to a coronary event^11–13^.

Thus, in this study, we aimed at defining the antigenic and functional profile of the two dominant MDM morpho-phenotypes in acute myocardial infarction (AMI) and in stable CAD disease patients, as compared to healthy subjects. Moreover, we evaluated whether these *in vitro* information reflected the *in vivo* morphology features of coronary plaques along with their macrophage content in acute and chronic CAD patients undergoing OCT assessment.

## Results

### Clinical features of subjects

Of the 90 consecutive CAD patients (age 65 ± 12 years, males 71), 41 (46%) had a diagnosis of SA, whereas 49 (54%) of AMI (28 NSTEMI [57%] and 21 STEMI [43%]). Table 1 shows the clinical and angiographic data. For comparison, 25 subjects without CAD history were also included (age 49 ± 15 years, males 10). OCT data concerning the investigated lesion of CAD patients according to their clinical presentation (SA vs. AMI patients), are reported in Supplementary Table 1.

**Table 1:** Baseline clinical, laboratory and angiographic characteristics of coronary artery disease patients according to their clinical presentation.

Variables	SA, *N* = 41	AMI, *N* = 49	*P* value
**Demographics**			
Age (years)	68 ± 9	62 ± 12	0.02
Male sex, n (%)	33 (81)	38 (77)	0.73
BMI (kg/m²)	28.6 ± 3.1	29.2 ± 4.1	0.45
**Clinical characteristics**			
Current smoker, n (%)	26 (63)	30 (61)	0.83
Diabetes mellitus, n (%)	11 (27)	23 (47)	0.05
Dyslipidemia, n (%)	21 (51)	26 (49)	0.86
Hypertension, n (%)	24 (58)	27 (55)	0.74
Family history of CAD, n (%)	19 (46)	27 (55)	0.41
LVEF (%)	55 ± 7	47 ± 6	0.45
**Laboratory data**			
WBC (×10^9^/l)	8.77 ± 3.04	9.21 ± 3.60	0.53
RBC (×10^12^/l)	4.62 ± 0.70	5.44 ± 3.59	0.15
Neutrophil count (×10^9^/l)	6.41 ± 3.21	5.96 ± 2.82	0.48
Lymphocyte count (×10^9^/l)	2.03 ± 0.83	1.95 ± 0.82	0.62
Eosinophil count (×10^9^/l)	0.37 ± 1.14	0.36 ± 1.08	0.94
Monocyte count (×10^9^/l)	0.63 ± 0.23	0.60 ± 0.25	0.56
Basophil count (×10^9^/l)	0.007 ± 0.03	0.006 ± 0.02	0.78
Platelets (×10^9^/l)	220 ± 74	227 ± 74	0.67
hs-CRP (mg/l)	2.10 (1.40–3.20)	13.20 (5.00–26.00)	0.0001^†^
Creatinine (mg/dl)	1.02 ± 0.37	0.96 ± 0.31	0.09
Glycaemia (mg/dl)	124 ± 27	131 ± 36	0.33
Total cholesterol (mg/dl)	170 ± 35	206 ± 45	0.0001
LDL (mg/dl)	97 ± 26	129 ± 41	0.0001
HDL (mg/dl)	50 ± 15	43 ± 10	0.009
Triglycerides (mg/dl)	122 ± 55	163 ± 63	0.002
Peak TnI (μg/dl)	0.001 (0.001–0.002)	12.30 (1.40–34.60)	0.0001^†^
Peak CK-MB (μg/dl)	2.10 (1.60–3.42)	85.40 (13.20–213.00)	0.0001^†^
**Angiographic data**			
Culprit or treated vessel, n (%)			0.03*
LAD	16 (39)	33 (67)	
LCX	7 (17)	5 (10)	
RCA	18 (44)	11 (23)	
Multivessel disease, n (%)	28 (68)	23 (47)	0.04
**Admission therapy**			
ASA, n (%)	14 (34)	20 (29)	0.65
Beta-Blockers, n (%)	12 (29)	14 (29)	0.94
ACE-inhibitors, n (%)	21 (51)	11 (22)	0.004
Statins, n (%)	17 (41)	14 (29)	0.2

Data are expressed as mean ± SD or median and interquartile range. *P* value: Wilcoxon test for quantitative variables. *Fisher test, ^†^Kruskal-Wallis test.

SA, stable angina; AMI, acute myocardial infarction; CAD, coronary artery disease; BMI, body-mass index; LVEF, left ventricular ejection fraction; WBC, white blood cells; RBC, red blood cells; LDL, low-density lipoprotein; HDL, high-density lipoprotein; hs-CRP, high-sensitive C-reactive protein; TnI, troponin-I; CK-MB, Creatine phosphokinase-MB; LAD, left anterior descending; LCX, left circumflex; RCA, right coronary artery; ASA, aspirin; ACE-inhibitors, angiotensin-converting-enzyme inhibitors.

### Biological profile of monocytes and MDMs

The distribution of classical and non-classical monocyte subsets in healthy subjects and in CAD patients was not different (CD14^++^CD16^−^ or classical monocytes: 85.77 ± 8.58% and 85.07 ± 7.25%; *P* = 0.667; CD14^+^CD16^++^or non-classical monocytes: 4.62 ± 2.2% and 3.69 ± 2.94%; *P* = 0.121, in healthy subjects and in CAD patients, respectively). An increase in intermediate monocytes (CD14^++^CD16^−^) count was detected in patients with CAD (10.46 ± 4.50% and 7.63 ± 1.95% in CAD patients and in healthy subjects, respectively; *P* = 0.001).

After *in vitro* differentiation of monocytes, MDMs positive for CD14 were 88.48 ± 5.64% and 87.80 ± 8.60% (*P* = 0.69) in healthy subjects and CAD patients, respectively. The CD14^+^CD16^+^ MDMs were 54.03 ± 19.84% and 52.60 ± 21.19% (*P* = 0.92) in healthy subjects and CAD patients, respectively.

### Morphological characterization of MDMs

**Figure 1:**
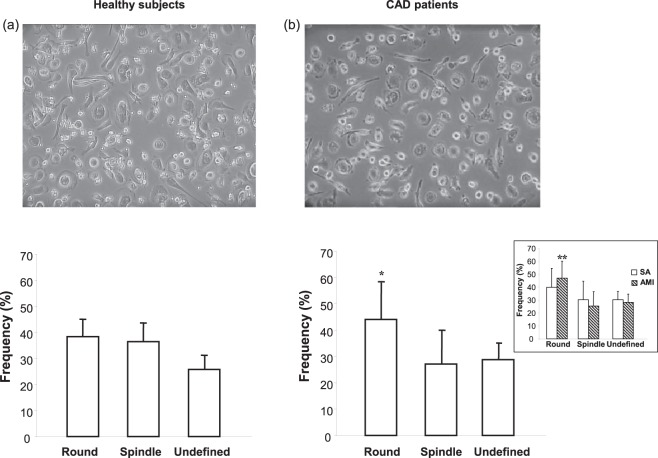
MDM morphology and frequency. (**a** and **b**) Representative image (AxioVert 200 M, Zeiss, 200× original magnification) of MDMs obtained from healthy subjects and CAD patients (top); frequency in round, spindle, and undefined MDMs in healthy subjects and in CAD patients (bottom). Inset, frequency in round, spindle, and undefined MDMs in stable angina (SA) and acute myocardial infarction (AMI) patients. Data are expressed as the mean ± SD, and they derive from independent cultures obtained from 25 healthy subjects and 50 CAD patients (19 SA and 31 AMI patients). *P < 0.001 round versus spindle and versus undefined MDMs, **P < 0.0001 round versus spindle and versus undefined MDMs.

MDMs of CAD patients had two predominant and different morphotypes, round and spindle. Differently from that observed in healthy subjects, who are characterized by a similar prevalence of these two morphotypes, in CAD patients, the prevalence of round MDMs was significantly higher than that of spindle or undefined MDMs (Fig. 1a,b). Furthermore, the frequency of round MDMs was particularly higher than that of spindle MDMs in AMI patients (Fig. 1 inset).

Both MDM morphotypes obtained from healthy subjects^10^ and CAD patients expressed the CD68 macrophage marker (data not shown).

### Efferocytic capacity of MDMs and levels of CD14 receptor and TG-2 protein

**Figure 2:**
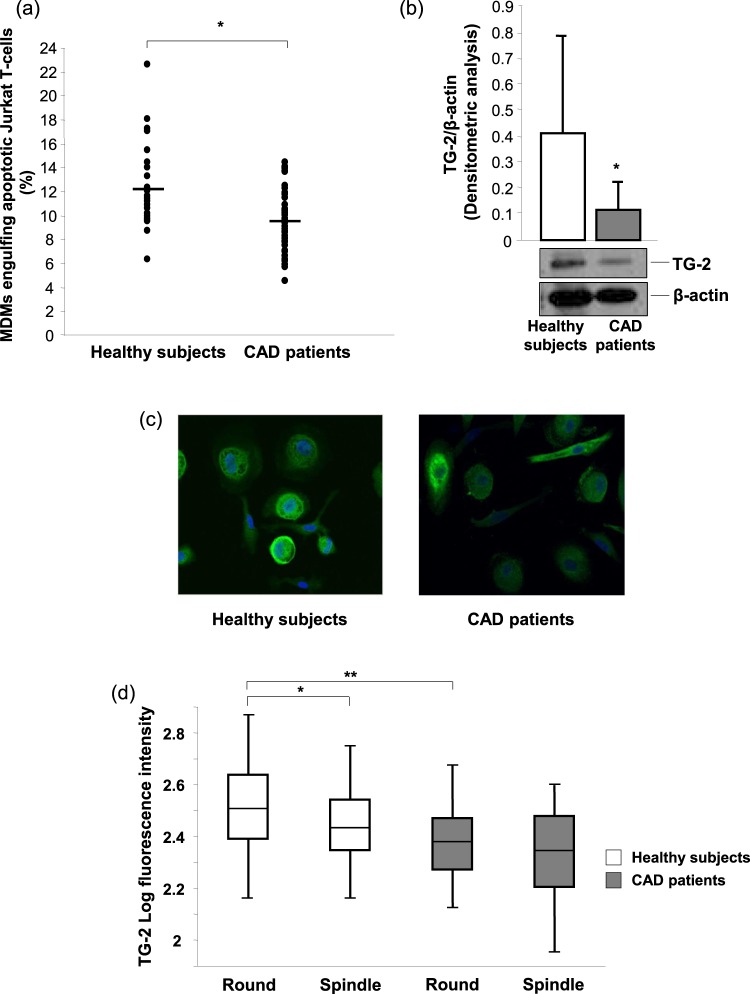
Efferocytosis and TG-2 levels in MDMs. (**a**) MDMs were co-cultured with early apoptotic Jurkat T-cells for 30 minutes, and the uptake was assessed by flow cytometry. Data are expressed as the percentage of MDMs that had engulfed carboxy fluoroscein diacetate succinimidyl ester (CSFE)-stained Jurkat apoptotic cells, and they derive from independent cultures obtained from 25 healthy subjects and 50 CAD patients. (**b**) TG-2 was detected by western blot analysis. β-actin was used as a control of protein loading. Densitometry is shown in the bar graph. Data are expressed as mean ± SD, and they derive from MDMs obtained from 5 healthy subjects and 10 CAD patients. (**c**) Representative images (400× original magnification) and (**d**) quantitative analysis of TG-2 in round and spindle MDMs obtained from healthy subjects and CAD patients. Nuclei were visualized by Hoechst 33258. Data derive from independent cultures obtained from 10 healthy subjects and 30 CAD patients. *P < 0.05, **P < 0.005. Full blots are shown in Supplementary Fig. 1.

The efferocytic capacity of the MDMs of CAD patients, either SA or AMI (9.0 ± 1.96% and 9.90 ± 2.65% of MDMs that had engulfed apoptotic Jurkat T-cells, respectively; *P* = 0.21), was lower than that of healthy subjects’ MDMs (Fig. 2a).

Efferocytosis comprises the recognition, tethering, and uptake of apoptotic cells, and several factors are involved in these processes. CD14 is a member of a complex recognition system utilized by macrophages for the clearance of apoptotic lymphocytes^14^. However, we found that the count of CD14-positive MDMs (88.11 ± 5.46% and 90.26 ± 5.08% in healthy subjects and in CAD patients, respectively; *P* = 0.09) and the Mean Fluorescence Intensity for CD14 receptor (36.80 ± 23.40 and 38.80 ± 19.10 in healthy subjects and in CAD patients, respectively; *P* = 0.73) was similar between healthy subjects and CAD patients.

Considering the relevant role of transglutaminase (TG)-2 in the formation of an efficient phagocyte portal in macrophages^15^, we verified whether changes in the expression of this protein may be responsible for the impaired efferocytosis of CAD MDMs. MDMs obtained from CAD patients had markedly lower levels of TG-2 than those obtained from healthy subjects (Fig. 2b). Of note, in healthy subjects, TG-2 levels were higher in round MDMs (Fig. 2c,d), the morphotype with a greater capacity to bind/uptake apoptotic cells^10^. In contrast, in CAD patients, TG-2 levels were similar in round and spindle MDMs, suggesting a potential contribution of this protein in the reduction of the apoptotic cell clearance detected in these patients (Fig. 2c,d).

### Levels of CD206 and CD163 receptors

**Figure 3:**
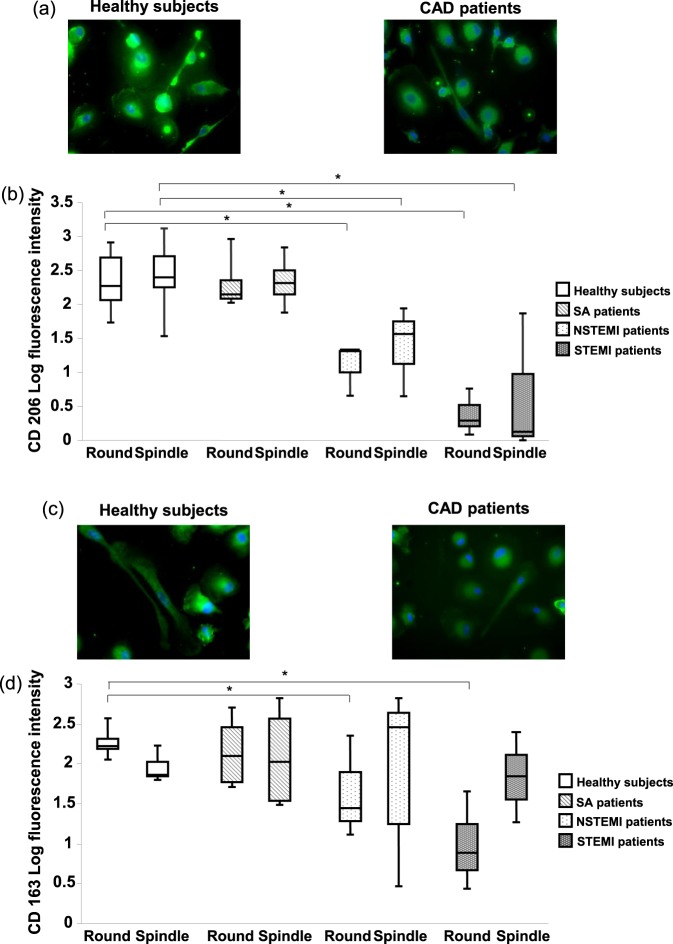
CD206 and CD163 levels in MDMs. (**a**) Representative images (400× original magnification) of CD206 in MDMs obtained from healthy subjects and CAD patients. (**b**) Quantitative analysis of CD206 levels in MDM morphotypes of healthy subjects and patients. (**c**) Representative images (400× original magnification) of CD163 in MDMs obtained from healthy subjects and CAD patients. (**d**) Quantitative analysis of CD163 levels in MDM morphotypes of healthy subjects and patients. Nuclei were visualized by Hoechst 33258. Data derive from independent cultures obtained from 3 healthy subjects, 3 SA patients, 3 NSTEMI patients, and 3 STEMI patients. *P < 0.05.

The mannose receptor CD206 and the haptoglobin/hemoglobin scavenger receptor CD163 have been associated with the non-inflammatory, anti-atherogenic macrophage phenotype^16,17^. A significant decrease of CD206 levels in both MDM morphotypes has been observed in NSTEMI and STEMI patients as compared to healthy subjects (Fig. 3a,b). In addition, CD163 levels were significantly lower only in the round MDM morphotype of NSTEMI and STEMI patients when compared to healthy subjects (Fig. 3c,d).

### Tissue factor levels and thrombin generation

**Figure 4:**
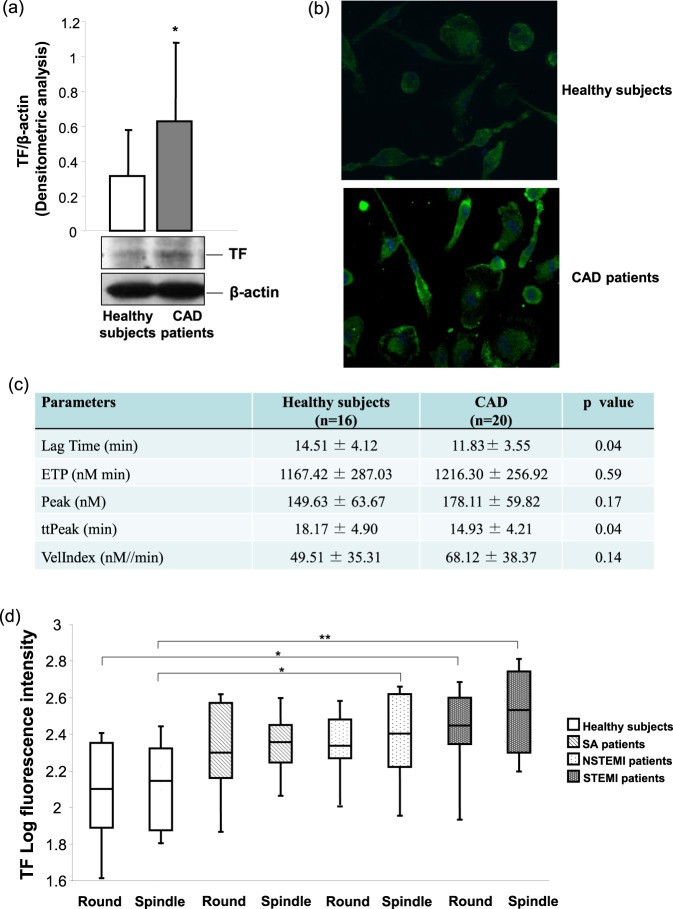
TF levels and thrombin generation in MDMs. (**a**) TF was detected by western blotting. β-actin was used as a control of protein loading. Densitometry is shown in the bar graph. Data are expressed as mean ± SD and derive from MDMs obtained from 12 healthy subjects and 14 CAD patients. (**b**) Representative images (400× original magnification) of TF in MDMs obtained from healthy subjects and CAD patients. Nuclei were visualized by Hoechst 33258. Data derive from independent cultures obtained from 10 healthy subjects and 30 CAD patients. (**c**) Thrombin generation parameters measured in MDMs obtained from 16 healthy subjects and 20 CAD patients. (**d**) Quantitative analysis of TF levels in MDM morphotypes of healthy subjects and patients. Data derive from independent cultures obtained from 10 healthy subjects, 10 SA patients, 10 NSTEMI patients, and 10 STEMI patients. *P < 0.05, **P < 0.005. Full blots are shown in Supplementary Fig. 2.

Tissue factor (TF) represent the main cellular activator of the blood coagulation cascade, allowing the thrombin generation. The MDMs of CAD patients had a significantly greater TF expression than those of healthy subjects (Fig. 4a). Moreover, a significantly greater TF fluorescence intensity was detected in both MDM morphotypes of CAD patients when compared to their counterparts in healthy subjects (Fig. 4b). This enhanced antigenic expression of TF displayed a functional consequence in thrombin generation. Indeed, the lag time and the time to peak in thrombin generation were lower in MDMs of CAD patients as compared to healthy subjects. In contrast, the peak of thrombin generation and the endogenous thrombin potential (ETP) did not change (Fig. 4c). Furthermore, an increasing gradient in TF levels going from MDMs (spindle and round) of healthy subjects to MDMs of SA, NSTEMI, and STEMI patients was observed. Of note, the highest TF levels were detected in both morphotypes of STEMI patients (Fig. 4d), suggesting a potential greater MDM contribution to thrombus formation in patients experiencing an acute coronary event.

### Matrix metalloproteinase-9 activity

**Figure 5:**
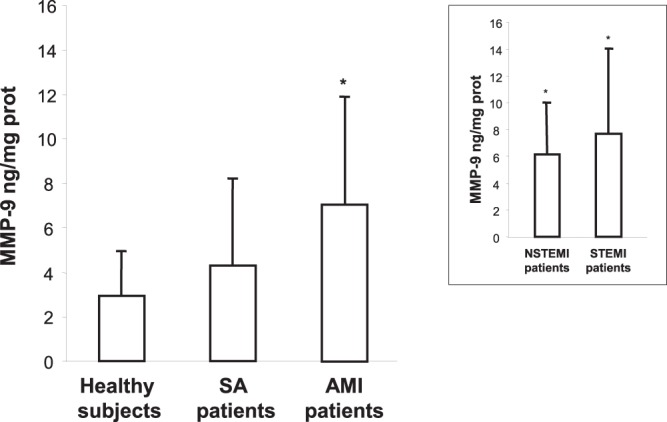
MMP-9 activity. MDMs were incubated overnight in medium containing 0.2% fatty-acid-free albumin. The activity of MMP-9 secreted by MDMs was evaluated by the Biotrak^TM^ activity assay system. Data are expressed as mean ± SD and derive from independent cultures obtained from 9 healthy subjects, 9 SA patients, and 18 AMI (9 NSTEMI and 9 STEMI) patients. *P < 0.05 vs. healthy subjects.

MDMs showed a progressive matrix metalloproteinase-9 (MMP-9) activity increase in SA or AMI patients, reaching a maximum in STEMI patients, as compared with healthy subjects (Fig. 5), suggesting a greater *in vivo* potential for MDMs to induce plaque vulnerability or rupture of the fibrous cap.

### MDM profile, angiographic analyses, and OCT plaque features

**Figure 6:**
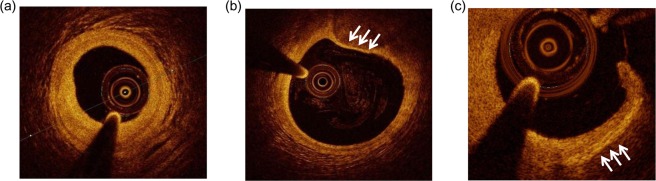
Representative OCT images of coronary atherosclerotic plaque. (**a**) Stable fibrous coronary plaque. (**b**) High-risk thin-cap fibroatheroma (white arrows). (**c**) Coronary rupture plaque with macrophage infiltration (white arrows).

Representative OCT images of coronary atherosclerotic plaques are showed in Fig. 6.

**Figure 7:**
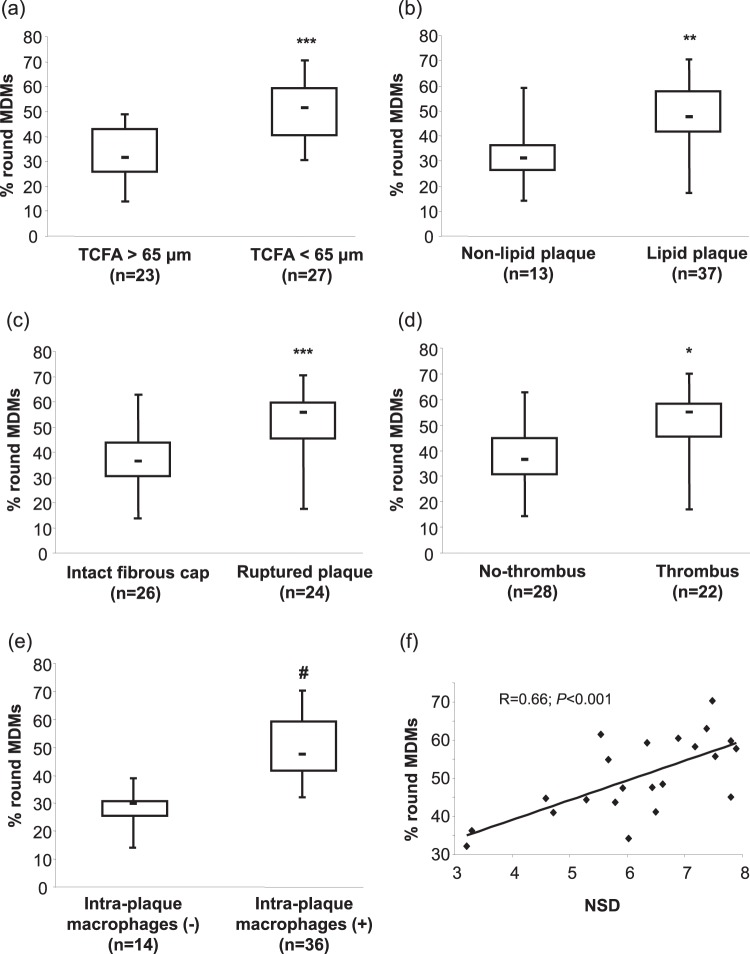
OCT plaque features and frequency of round MDM morphotype. Correlation between round MDM morphotype and (**a**) thin-cap fibroatheroma (TCFA), (**b**) plaque lipid content, (**c**) fibrous cap integrity, (**d**) presence of thrombi, (**e**) presence of intra-plaque macrophages, and (**f**) macrophage normalized standard deviation (NSD), detected by means OCT in *in vivo* plaque. *P < 0.02, **P < 0.002, ***P < 0.0005, ^#^*P* < 0.0001.

The prevalence of *in vitro* round MDMs was significantly higher in patients presenting a thin-cap fibroatheroma (TCFA), a lipid plaque, a ruptured plaque, and a thrombus (Fig. 7a–d). The prevalence of round MDMs inversely correlated with plaque cap thickness (R = −0.65; *P* < 0.0001), and it positively correlated with the maximum lipid quadrant (R = 0.36; *P* = 0.04). The quantification of CAD severity can be captured using coronary angiography, and several scoring systems were developed to assess it. At angiographic analysis of CAD severity, round MDMs tended to correlate with the Bogaty extent (R = 0.35; *P* = 0.08) and severity (R = 0.37; *P* = 0.07) scores, and they significantly correlated with the Sullivan score (R = 0.57, *P* = 0.002). Moreover, the intra-plaque macrophages identification was associated with a higher round MDMs prevalence (Fig. 7e), and the latter positively correlated with normalized standard deviation (NSD) (R = 0.66; *P* < 0.001; Fig. 7f).

Moreover, the MDM efferocytic ability positively correlated with the minimal lumen area (MLA) (R = 0.49; *P* = 0.013), and it tended to inversely correlate with stenosis plaque percentage (R = 0.35; *P* = 0.08). In addition, when evaluating indexes of angiographic CAD severity, MDM efferocytic capacity inversely correlated with the Bogaty extent (R = −0.43; *P* = 0.04) and severity (R = −0.41; *P* = 0.05) scores, and with the Sullivan score (R = −0.47; *P* = 0.04).

However, TG-2 in round MDMs and in spindle MDMs did not correlate with MLA (*P* = 0.50; *P* = 0.21) nor with stenosis plaque percentage (*P* = 0.91; *P* = 0.32).

TF levels in round and spindle *in vitro* obtained MDMs positively correlated with the *in vivo* detection of a ruptured plaque (R = 0.36, *P* = 0.05 and R = 0.47, *P* = 0.01, respectively). Additionally, TF expression in both MDM morphotypes positively correlated with the presence of thrombus (R = 0.36, *P* = 0.05 and R = 0.47, *P* = 0.01, respectively). Finally, TF levels in round and spindle MDMs positively correlated with the intra-plaque macrophages presence (R = 0.57, *P* = 0.001 and R = 0.64, *P* < 0.001, respectively), and they tended to positively correlate with NSD (*P* = 0.08 and *P* = 0.11).

All these observations may suggest that the profile of MDMs may be related to high-risk features of the coronary plaque morphology and activity.

## Discussion

In this report, we show for the first time that the spontaneous *in vitro* differentiation of monocytes into MDMs of CAD patients results in a higher prevalence of round MDMs, having a lower efferocytic capacity and an enhanced thrombin generation capacity, as compared to healthy subjects. Additionally, in CAD patients, the propensity of monocytes to differentiate into round MDMs is associated with the detection of vulnerable and/or ruptured plaque, as assessed by OCT intracoronary imaging.

Studies on tissue-resident macrophages are very challenging due to the difficulties in macrophage extraction and tissue availability. Moreover, coronary atherosclerotic plaque is a compartment not easily accessible, and macrophages obtained from a spontaneous differentiation of circulating monocytes may be considered a good surrogate. By investigating MDM morpho-phenotypes in the different CAD clinical presentation, we found that, differently from healthy subjects, who are characterized by a similar frequency of spindle and round cells^10^, CAD patients have mostly round MDMs. This was unexpected, as round MDMs in healthy subjects proved to exhibit anti-inflammatory properties. However, the differences between CAD patients and healthy subjects in terms of MDMs are not confined to morphology, but also reflected in different antigenic and functional profiles. In particular, despite the prevalence of round cells, MDMs of CAD patients were characterized by an anti-inflammatory properties reduction as compared to MDMs of healthy subjects, as supported by their lower levels of CD206 and CD163, both markers of the anti-inflammatory macrophage phenotype^16,17^. Indeed, it has been shown that macrophages expressing high levels of CD206 and CD163 had a reduced pro-inflammatory cytokine production^18^; and, accordingly, a high expression of CD206 was peculiar of phagocytic macrophages^19^. Therefore, as plaque development may originate also from inadequate anti-inflammatory responses, the macrophage polarization imbalance may play a critical role in plaque formation^20,21^. Notably, macrophage phenotype is affected by the surrounding microenvironment and by cell-cell interactions. Indeed, the interaction between CD40L (Ligand), expressed on T lymphocytes surface, and CD40, highly expressed in pro-inflammatory macrophages^22^, induces the expression of adhesion molecules, and the release of matrix metalloproteinases and pro-inflammatory cytokines, promoting monocyte recruitment and plaque progression^23^. Thus, the imbalance in the inflammatory response in atherosclerotic plaques might be supported by a loss of anti-inflammatory properties of the round MDMs detected in CAD patients. To further investigate this observation, we evaluated the efferocytic capacity of MDMs and correlated it with angiographic and OCT CAD burden. Interestingly, we found a significant reduction of the efferocytic ability in the MDMs of CAD patients as compared to healthy subjects, and, in our cohort of CAD patients, coronary angiographic analyses demonstrated that, the lower was the MDM efferocytic capacity, the more severe and extensive was the coronary atherosclerotic burden. A similar behavior was observed when considering MLA and stenosis grade of the investigated lesion by means of OCT. It is well known that, at coronary plaque level, apoptotic cell death and efferocytic rates are strongly related to atherosclerotic lesion stage^24^. The apoptotic cell clearance enhances the resolution of the ongoing inflammatory process, by modulating several and critical inflammatory pathways, including IL-10 and TGF-β release, and promoting phagocyte survival^25–27^. Thus, even a small reduction in efferocytic ability may have relevant clinical implications. The reduced efferocytosis detected in CAD MDMs did not depend on the binding of apoptotic cell mediated by the CD14 receptor, but it was related to the expression of TG-2, a protein-cross-linking enzyme involved in an efficient phagocytic portal formation^15^. These observations further confirm the presence of significant functional differences of CAD MDMs, as compared to healthy subjects, that may be involved in atherosclerosis initiation and progression and potentially in its complications. This may pave the way to pharmacological modulation studies aimed at limiting progression and improving stabilization of coronary atherosclerosis, by a direct targeting of specific MDM subpopulations. Of note, recently published studies found that antibodies blocking CD47, a key anti-phagocytic molecule, could restore phagocytosis and prevent atherosclerosis^28^.

Macrophages are intrinsically linked not only to atherosclerotic disease progression but also to disease activity. Indeed, the amount of monocyte-macrophages infiltrating the plaque and their detection at plaque rupture-sensitive sites are associated to plaque vulnerability^29,30^. Interestingly, we found that the propensity of circulating monocytes to differentiate into round MDMs in CAD patients was associated with a vulnerable plaque presence and with a ruptured plaque, as evaluated by OCT analyses. The risk of an acute coronary event is related to the composition and activity of the plaque, and inflammation is crucial for its instability^31–33^. Our observation could imply that round MDMs in CAD patients may reduce lesion integrity and increase the likelihood of an acute myocardial infarction. Indeed, the presence of a thinner fibrous cap, a larger lipid core, and a greater macrophage content were more frequently detected in CAD patients with a higher prevalence of *in vitro* round MDMs. Thus, although our data provide initial observational insights on the potential association between vulnerable coronary plaques and round MDMs in CAD patients, this morpho-type might represent a tool able to recognize high-risk patients of developing an acute myocardial infarction. Yet, future mechanistic studies and/or transcriptomic investigations, combined with the *in vivo* analysis of coronary atherosclerotic plaques, are needed in order to support our findings, to identify the involved signal transduction, and to explore the contribution of an intrinsic differentiation program or of environmental imprinting of monocytes, in determining the diversity of MDMs^34^.

Finally, it is known that plaque macrophage accumulation may favor both rupture of fibrous cap, due to pro-inflammatory cytokines and proteolytic enzymes secretion, and thrombus formation, due to TF release^35^. It has been shown that circulating TF may potentiate the thrombogenic stimulus and upregulate the inflammatory response^36,37^. TF is synthetized by macrophage-derived foam cells in atherosclerotic plaques, and its expression is increased in lipid-rich plaques^38,39^. In our report, the MDMs obtained from AMI patients showed a higher TF fluorescence than the MDMs of healthy subjects, with a peak in STEMI patients. In addition, we found that TF levels in spindle and in round MDMs positively correlated with the OCT detection of a ruptured plaque and of thrombi. Accordingly, it has been documented that TF levels were higher in atheroma of patients with acute coronary syndrome than with SA^40^. Then, TF expression may enhance the severity of acute coronary thrombosis, further highlighting the functional differences between the MDMs of CAD patients and those of healthy subjects. Indeed, round MDMs in CAD patients seem to lose their anti-inflammatory properties, and to acquire pro-thrombotic features, potentially contributing, at least in part, to acute coronary event initiation and severity. The mechanisms underlying this observation cannot be deduced from our study; yet, this study shows a peculiar phenotype of round MDMs in CAD patients that may potentially affect the inflammatory status and the pathogenesis of coronary atherosclerosis and atherothombosis.

Our findings might have some potential clinical implications. Although this is an observational study and a cause-effect relationship cannot be established, the definition of an MDM profile may help to predict CAD burden and coronary plaque composition, also contributing to the differentiation between stable and unstable plaques. Moreover, the development of therapies that blunt macrophage cytotoxicity, plaque growth, and destabilizing functions and enhance their natural reparative properties, may represent a new approach to curtail macrophage-mediated injury, limit coronary stenosis progression and enhance plaque stability^41^, the two most important aims when counteracting coronary atherosclerosis. Of interest, emerging evidences suggest the possibility of pharmacologically modulating the functions of macrophages^42–44^. In particular, our findings, if confirmed in larger study populations, may pave the way to novel diagnostic tools, able to early identify patients at high risk of severe CAD and/or of atherothrombosis. Further, our observations may be useful to develop novel therapies able to manipulate macrophage morpho-phenotype, with the ultimate goal of reducing CAD burden and first or recurrent acute cardiovascular events.

Some limitations warrant mention. First, the small sample size of our study limits the general applicability of our findings. Second, even if the macrophage heterogeneity has been documented in tissues, these macrophage phenotypes may not correspond to phenotypes generated *in vitro*. Indeed, in atherosclerotic lesions, macrophages respond to several environmental stimuli, such as cytokines and modified lipids, and they interact with other cells, including erythrocytes, lymphocytes, and platelets^45–47^, potentially modifying their phenotypes. Thus, an assessment of monocyte functions and of their differentiation process via interaction with other cells, such as lymphocytes and platelets, and in relation to progenitors and stem cells, is needed. Third, our *in vitro* model may be used as a representative model of tissue macrophages^10,48,49^, however, the lack of cell turnover and of tissue-specific matrix proteins, are crucial in tissue macrophage behavior. Fourth, OCT imaging is a suboptimal reflection of true histology, mainly for macrophage infiltration^50,51^.

In conclusion, we demonstrated that the MDMs of CAD patients, as compared to healthy subjects, show a peculiar morpho-phenotype profile, which is characterized by a predominance of round MDMs, with reduced anti-inflammatory properties and a higher propensity for thrombogenicity than those of healthy subjects. Notably, this specific profile is associated with the detection of high-risk and rupture-prone coronary plaques, at OCT investigation. Assessing the functional features of macrophage phenotypes may allow to shed lights on their contribution to coronary atherosclerosis, thus providing novel diagnostic, therapeutic, and, more importantly, preventative regimens for CAD.

## Methods

### Study design

The study was carried out at Centro Cardiologico Monzino, Milan, Italy. Ninety consecutive CAD patients undergoing coronary angiography due to stable angina (SA) or AMI, as their first ischemic heart disease event, and showing obstructive atherosclerosis (>50% diameter stenosis by visual estimate) were enrolled. Twenty-five healthy subjects, with neither history of CAD, nor cardiovascular risk factors, nor inflammatory disorders, and specifically not taking any cardiovascular therapy, were recruited as control group.

In the initial 40 CAD patients, we performed the characterization of MDMs of CAD patients, and we compared these data to those obtained in healthy subjects. Subsequently, we evaluated the association between this profile and CAD clinical presentation, and we also assessed plaque composition in 50 patients undergoing OCT evaluation.

The study was approved by the institutional Ethic Committee (Comitato Etico IRCCS-Istituto Europeo di Oncologia e Centro Cardiologico Monzino), and it was performed according to the Declaration of Helsinki. All the study subjects provided written informed consent at the time of enrollment.

### Culture of MDMs

MDMs were obtained from a differentiation of monocytes isolated from venous blood of CAD patients and healthy subjects as described^10^. Briefly, mononuclear cells were obtained by density centrifugation on Ficoll-Paque (GE Healthcare, EuroClone, Milan, Italy) and plated (2 × 10^6^/mL) in 35 mm well plates (Primaria^TM^, Falcon, Sacco S.r.l, Como, Italy). After 90 minutes, non-adherent cells were removed, and adherent monocytes were cultured over 7 days in Medium 199 (Lonza, EuroClone, Milan, Italy) supplemented with 2 mM L-glutamine, 100 U/ml penicillin, 100 μg/ml streptomycin, and 10% autologous serum freshly obtained from blood clotted for 2 hours at 37 °C. Medium was not replaced throughout the culture period. The morphology of MDM was inspected by phase contrast microscopy (AxioVert 200 M, Zeiss, Milan, Italy) at 20× or 40× magnification. MDMs were defined spindle when a length >70 µm and a width <30 µm were detected, and round when width and length were similar and >35–40 µm. Cells of morphology and/or dimension that did not meet the above criteria were classified as undefined^10^.

### Flow cytometric analysis of monocytes and MDMs

Monocyte subsets were analyzed in 100 μl of whole blood collected from all participants. Adherent MDMs were detached with trypsin.

Cells were stained for 10 min with monoclonal mouse anti-human CD14 APC-conjugated and CD16 FITC-conjugated antibodies (BD Biosciences, Milan, Italy) and analyzed using flow cytometry^52^. Data were analyzed using CellQuest analysis software (Becton-Dickinson, Oxford, UK).

### Apoptosis of Jurkat T-cells

Jurkat T-cell line, clone E6-1 (ATCC), was purchased from LGC Standards S.r.l. (Milan, Italy), and cultured in RPMI 1640 supplemented with 10% fetal calf serum (Lonza, EuroClone) and 26 mM HEPES. Early apoptosis was induced by incubating cells (4 × 10^6^ cells/ml) for 2 hours in serum-free RPMI 1640 with 10 µM etoposide (Sigma Aldrich, Milan, Italy)^53^. The extent of apoptosis was evaluated by flow cytometry using annexin V PE (BD Biosciences), according to the manufacturer’s instructions, and 20,000/sample events were acquired.

### Efferocytosis assay

Before the induction of apoptosis, Jurkat T-cells were stained with 5 µM carboxyfluorescein diacetate succinimidyl ester (CFSE, Life Technologies, Milan, Italy) for 30 minutes at 37 °C. CSFE-labeled apoptotic cells (1 × 10^6^ cells/ml) were co-cultured with MDMs (2:1 ratio) for 30 minutes. Non-ingested cells were removed. Adherent MDMs were detached with trypsin, incubated with APC anti-human CD14 antibody (BD Biosciences), or isotype-matched irrelevant antibody, for 15 minutes at room temperature (RT), and analyzed by flow cytometry. 10,000 events were acquired, and the data were analyzed using CellQuest software (Becton-Dickinson). Data are expressed as percentage of CD14-positive MDMs that had binding/engulfed CSFE-labeled apoptotic Jurkat T-cells^54^.

### Western blot analysis

Western blotting was performed as previously described^55^. Nitrocellulose blotting membranes were incubated with primary antibodies directed against TG-2, (Cell Signaling, EuroClone, Milan, Italy), or TF (American Diagnostica, Sekisui Diagnostics, Cabru, Milan, Italy). After incubation with a horseradish peroxidase-conjugated secondary antibody (Jackson ImmunoResearch Labs Inc., Li StarFISH, Milan, Italy), the immunoreactive protein bands were detected by chemiluminescence. β-actin was used as loading control.

### Thrombin generation measurement

MDMs were lysed in 15 mM octyl-$\beta$-D-glycopiranoside (Sigma Aldrich) as previously described^56^. One µg lysed cells (20 µl) were mixed with 10 µl of 25 mM HEPES buffered-saline and 20 µl of platelet-poor plasma. Thrombin generation was triggered by the addition of fluorogenic substrate Z-Gly-Gly-Arg-AMC (Stago Italia S.R.L. Unipersonale, Milan, Italy) in the presence of CaCl_2_ and measured for 60 minutes using the Calibrated Automated Thrombogram^®^ assay (Thrombinoscope BV, Maastricht, the Netherlands). The lag-time, peak of thrombin generation, ETP, and velocity index of propagation phase (VelIndex) were calculated using Thrombinoscope software (Thrombinoscope BV).

### Immunofluorescence staining

MDMs were fixed (2% para-formaldehyde, for 20 min at RT), and non-specific reactive sites were blocked with 5% bovine serum albumin (BSA) solution containing 0.1% saponin (30 minutes, RT)^10^. MDMs were incubated overnight at 4 °C with antibodies directed toward CD68 (Dako Italia S.p.A., Milan, Italy), TG-2, TF, CD206 (Abcam, Prodotti Gianni, Milan, Italy), CD163 (AbD serotec, Space Import-Export, Milan, Italy). Detection was performed with appropriate Alexa Fluor 488 (60 minutes, RT) (Life Technologies). Negative control were performed in parallel by omitting the primary antibodies.

### Quantitative fluorescent image analysis

Digital images were taken on an AxioObserver.Z1 microscope connected to a camera, and processed using the AxioVision 4.7 (Zeiss) software. Fluorescence intensity (densitometric sum of gray) was quantified, as an index of the amount of the protein investigated^10^. Data are expressed as log median of fluorescence intensity/μm^2^ and interquartile range for each MDM morphotype, subtracting the negative control value. Multiple fields of view (at least three fields, 400× magnification) were taken for each culture. Means derive from 10 independent cultures obtained from different donors.

### Matrix metalloproteinase-9 activity

MDMs were washed, and fresh medium containing 0.2% fatty acid free albumin was added. After overnight incubation, supernatant was collected and centrifuged, and the activity of MMP-9 secreted by MDMs was evaluated by the Biotrak^TM^ activity assay system (Amersham, GE Healthcare, Milan, Italy).

### Angiographic analyses

Angiographic analyses for the evaluation of CAD severity and extent were focused on the assessment of the Bogaty score^57^ and the Sullivan extent score^58^.

### OCT image analysis

Culprit lesion, in AMI patients, was identified by angiography, electrocardiographic T-wave or ST-segment modifications, and/or regional wall motion abnormalities at echocardiogram. In SA patients, OCT analysis was performed at the MLA site. Frequency domain OCT (FD-OCT) images were acquired by a commercially available system (C7 System, LightLab Imaging Inc/St Jude Medical, Westford, MA) connected to an OCT catheter (C7 Dragonfly; LightLab Imaging Inc/St Jude Medical), which was advanced to the culprit lesion. All images were digitally recorded and stored, and every single frame (0.2 mm) was analyzed by two independent investigators from an OCT core lab, who were blinded to clinical and laboratory values (Institute of Cardiology, Catholic University of the Sacred Heart, Policlinico Gemelli, Rome, Italy)^59^.

At the MLA site or at culprit lesion, respectively in SA and AMI patients, the analysis was targeted on plaque characterization (calcified, fibrous, or lipid plaques), presence of plaque rupture, measurement of fibrous cap thickness, and presence of intracoronary thrombi and intra-plaque microchannels, as previously described^11–13^. When a plaque contained two or more lipid-containing quadrants, it was considered a lipid-rich plaque, and the lipid arc and the cap thickness were measured. TCFA was defined as a lipid-rich plaque with a fibrous cap thickness of ≤65 µm^60^.

### OCT macrophage analysis

The presence of macrophage infiltration (MØI) in the lesions analyzed by OCT was assessed as previously reported^59^. Briefly, macrophages were qualitatively identified on raw OCT data within a 300 × 125 µm^2^ (lateral × axial) region of interest (ROI), according to the International Working Group for Intravascular Optical Coherence Tomography (IWG-IVOCT) Consensus standards^12^.

Macrophages were visualized as signal-rich, distinct, or confluent punctate regions exceeding the intensity of background speckle noise and generating a backward shadowing. For caps having a thickness <125 µm^2^, the depth of the ROI was matched to the cap thickness. Median filtering was performed with a 3 × 3 square kernel to remove speckle noise. In plaques with MØI, quantitative evaluation of macrophage content was obtained by measuring the NSD known to have a high degree of positive correlation with histological measurements of macrophage content, by using a dedicated software provided by S. Jude Medical^13,61^. NSD was measured for each pixel within each cap using a 125 µm^2^ window centered at the pixel location: NSD (x, y) = [σ (x, y)125 µm^2^/(Smax-Smin)] × 100, where NSD (x, y) is the normalized standard deviation of the OCT signal at pixel location (x, y), Smax is the maximum OCT image value, and Smin is the minimum OCT image value. Pixels within the (125 × 125) µm^2^ window that did not overlap with the segmented cap were excluded^13^.

### Statistical analysis

The distribution of continuous variables was assessed by visual inspection of frequency histograms and with the use of the Shapiro-Wilk test. Continuous variables were expressed as mean ± standard deviation (SD) or median with interquartile range, if they followed a normal or non-normal distribution, respectively. Continuous variables were compared with unpaired t-test or Mann-Whitney U-test, whereas categorical variables were compared using the Chi square test or Fisher’s exact test, as appropriate. Paired-group comparison was performed using the paired t-test or Wilcoxon test, as appropriate. Unpaired t-test or Mann-Whitney U-test were used for the comparison of continuous variables between groups; continuous variables among groups were compared with ANOVA or Kruskal-Wallis test, and Bonferroni’s correction for multiple comparison was applied and the Bonferroni-adjusted-*P*-value was reported. Correlations between variables were performed using the Pearson test or the Spearman’s rank test, as appropriate. Intra-observer and inter-observer variability in the analysis of MØI in the fibrous cap were assessed by Kappa measure of agreement. Regarding the evaluation of MØI, Kappa measures of agreement for intra-observer and inter-observer variability were 0.89 (*P* < 0.0001) and 0.94 (*P* < 0.001), respectively. All performed analyses were adjusted for age and sex.

All tests were two-sided. A *P* value < 0.05 was considered to indicate statistical significance. All calculations were computed with the aid of the SAS software package (Version 9.2; SAS Institute Inc., Cary, NC).

## Supplementary information

Supplementary information
