# Supplementary material for: Biological profile of monocyte-derived macrophages in coronary heart disease patients: implications for plaque morphology
Source: Sci Rep. 2019 Jun 18;9:8680. doi: 10.1038/s41598-019-44847-3 (PMC6581961; doi:10.1038/s41598-019-44847-3)
Supplement: Supplementary file 1 — Supplementary information [file 41598_2019_44847_MOESM1_ESM.pdf]

Supplementary Information

Biological profile of monocyte-derived macrophages in coronary heart disease patients: implications for plaque morphology

Sonia Eligini[#1,*], Nicola Cosentino[#1], Susanna Fiorelli[1], Franco Fabbiocchi[1], Giampaolo Niccoli[2], Hesham Refaat[2], Marina Camera[1,3], Giuseppe Calligaris[1], Stefano De Martini[1], Alice Bonomi[1], Fabrizio Veglia[1], Francesco Fracassi[2], Filippo Crea[2], Giancarlo Marenzi[1], and Elena Tremoli[1]

Author affiliation

[1] Centro Cardiologico Monzino I.R.C.C.S., Milan, Italy

[2] Department of Cardiovascular & Thoracic Sciences, Fondazione Policlinico Universitario A. Gemelli, I.R.C.C.S., Rome, Italy; Università Cattolica del Sacro Cuore, Istituto di Cardiologia, Rome, Italy

[3] Dipartimento di Scienze Farmacologiche e Biomolecolari, Università degli Studi di Milano, Milan, Italy

[#] These authors contributed equally to this work

Corresponding author:

Sonia Eligini, PhD,

Centro Cardiologico Monzino I.R.C.C.S.

Via Parea 4, 20138 Milan, Italy

Telephone: +39.02.58002839

Fax: +39.02.58002750

E-mail address: sonia.eligini@cardiologicomonzino.it

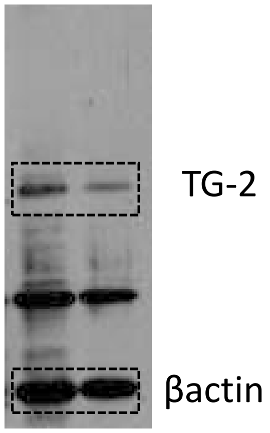

Supplementary figure 1. Full blot images of result shown in figure 2

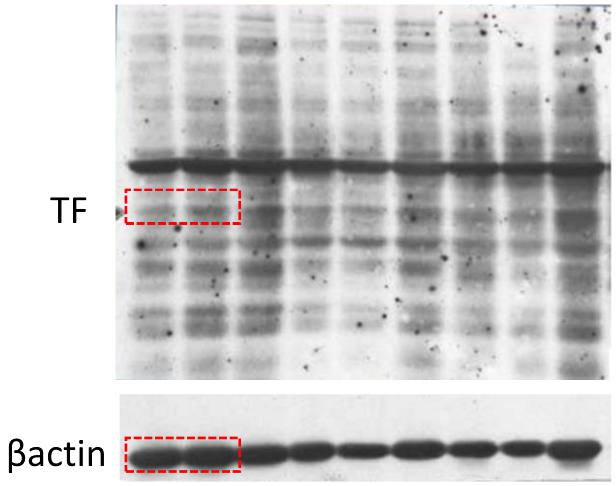

Supplementary figure 2. Full blot images of result shown in figure 4

Variables	SA, *N*=26	AMI, *N*=24	*P* value
Lipid plaque, n (%)	15 (57)	22 (92)	0.01
Fibrous plaque, n (%)	9 (35)	2 (8)	0.04*
Calcific plaque, n (%)	7 (27)	2 (8)	0.14*
Plaque rupture, n (%)	6 (23)	18 (75)	0.01
MLA, mm^2	1.59 (0.71-3.85)	1.54 (0.70-3.75)	0.99
TCFA, n (%)	8 (31)	19 (79)	0.001
Thrombus, n (%)	2 (8)	20 (83)	0.0001*
White, n (%)	1 (4)	18 (75)	0.0001*
Red, n (%)	1 (4)	7 (29)	0.02*
Lipid quadrants, n	2.1±1.0	3.1±1.6	0.0001
Lipid arc degree °	135 (87-265)	269 (161-280)	0.0001
Presence of microchannels, n (%)	4 (15)	15 (63)	0.0006*
Macrophage infiltration detection, n (%)	14 (54)	22 (92)	0.001
Macrophage NSD	3.45±1.01	6.19±1.95	0.001

Supplementary Table 1. Optical coherence tomography features of coronary artery disease patients according to the clinical presentation. Data are expressed as mean ± SD or median and interquartile range. p value: Wilcoxon test for quantitative variables; *Fisher test.

MLA, minimal lumen area; TCFA, thin-cap fibroatheroma; NSD, normalized standard deviation.
